# 2,5-Diphenyl­penta-2,4-dienenitrile

**DOI:** 10.1107/S160053680904481X

**Published:** 2009-10-31

**Authors:** R. Archana, A. Balamurugan, A. Manimekalai, A. Thiruvalluvar, R. J. Butcher

**Affiliations:** a PG Research Department of Physics, Rajah Serfoji Government College (Autonomous), Thanjavur 613 005, Tamil Nadu, India; b Department of Chemistry, Annamalai University, Annamalai Nagar 608 002, Tamil Nadu, India; c Department of Chemistry, Howard University, 525 College Street NW, Washington, DC 20059, USA

## Abstract

In the title compound, C_17_H_13_N, the dihedral angle between the two phenyl rings is 17.6 (1)°. An inter­molecular C—H⋯N hydrogen bond is found in the crystal structure, also a C—H⋯π inter­action involving the phenyl ring at position 5.

## Related literature

For the prebiotic synthesis of biological mol­ecules, see: Guillemin *et al.* (1998 ▶). For the preparation of flavonoid pigments, see: Fringuelli *et al.* (1994 ▶). For sexual pheromones, see: Liu *et al.* (1981 ▶). For the manufacture of light-emitting diodes (LEDs) with air-stable electrodes, see: Maruyama *et al.* (1998 ▶); Segura *et al.* (1999 ▶); Gómez *et al.* (1999 ▶).
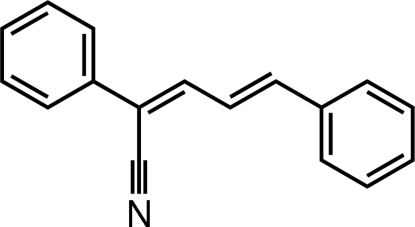


## Experimental

### 

#### Crystal data

C_17_H_13_N
*M* *_r_* = 231.28
Monoclinic, 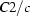

*a* = 16.9390 (5) Å
*b* = 7.5869 (2) Å
*c* = 19.3809 (6) Å
β = 99.521 (3)°
*V* = 2456.42 (13) Å^3^
*Z* = 8
Cu *K*α radiation
μ = 0.56 mm^−1^
*T* = 110 K
0.53 × 0.36 × 0.29 mm

#### Data collection

Oxford Diffraction Xcalibur Ruby Gemini diffractometer
Absorption correction: multi-scan (*CrysAlis Pro*; Oxford Diffraction, 2009 ▶) *T*_min_ = 0.713, *T*_max_ = 1.000
4517 measured reflections
2420 independent reflections
2322 reflections with *I* > 2σ(*I*)
*R*_int_ = 0.014

#### Refinement

*R*[*F* ^2^ > 2σ(*F* ^2^)] = 0.044
*wR*(*F* ^2^) = 0.121
*S* = 1.02
2420 reflections
163 parameters
H-atom parameters constrained
Δρ_max_ = 0.34 e Å^−3^
Δρ_min_ = −0.22 e Å^−3^

### 

Data collection: *CrysAlis Pro* (Oxford Diffraction, 2009 ▶); cell refinement: *CrysAlis Pro*; data reduction: *CrysAlis Pro*; program(s) used to solve structure: *SHELXS97* (Sheldrick, 2008 ▶); program(s) used to refine structure: *SHELXL97* (Sheldrick, 2008 ▶); molecular graphics: *ORTEP-3* (Farrugia, 1997 ▶); software used to prepare material for publication: *PLATON* (Spek, 2009 ▶).

## Supplementary Material

Crystal structure: contains datablocks global, I. DOI: 10.1107/S160053680904481X/wn2360sup1.cif

Structure factors: contains datablocks I. DOI: 10.1107/S160053680904481X/wn2360Isup2.hkl

Additional supplementary materials:  crystallographic information; 3D view; checkCIF report

## Figures and Tables

**Table 1:** Hydrogen-bond geometry (Å, °)

*D*—H⋯*A*	*D*—H	H⋯*A*	*D*⋯*A*	*D*—H⋯*A*
C54—H54⋯N13^i^	0.95	2.61	3.388 (2)	139
C56—H56⋯*Cg*1^ii^	0.95	2.83	3.657 (1)	146

Symmetry codes: (i) 
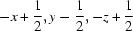
; (ii) 
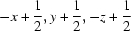
. *Cg*1 is the centroid of the C51–C56 phenyl ring.

**Fig. 1.**
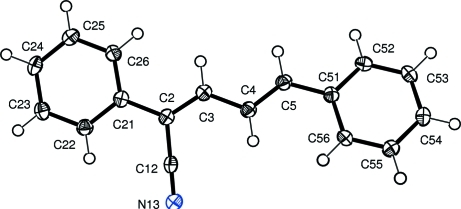
The molecular structure of the title compound, showing the atom-numbering scheme and displacement ellipsoids drawn at the 50% probability level. H atoms are shown as small spheres of arbitrary radius.

**Fig. 2.**
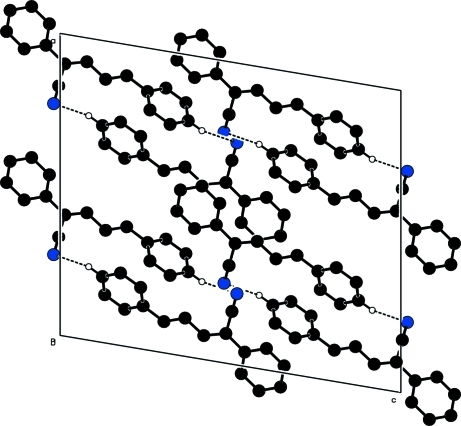
The packing of the title compound, viewed down the b axis. Dashed lines indicate hydrogen bonds. H atoms not involved in hydrogen bonding have been omitted.

## supplementary crystallographic information

### Comment

Unsaturated nitriles play a key role in many of the pathways proposed for the prebiotic synthesis of biological molecules (Guillemin *et al.,* 1998). Arylacrylonitriles are important synthons for the synthesis of several biologically active molecules in the preparation of flavonoid pigments (Fringuelli *et al.,* 1994) and sexual pheromones(Liu *et al.,* 1981). Recently arylacrylonitriles have been used to obtain high electron affinity polymers which can be used to manufacture light-emitting diodes (LEDs) with air-stable electrodes (Maruyama *et al.,* 1998; Segura *et al.,* 1999; Gómez *et al.,* 1999).

As part of our research, we have synthesized the title compound and report its crystal structure here. In the molecule, the dihedral angle between the two phenyl rings is 17.6 (1)°. The angle N13—C12—C2 is 178.90 (13)°, indicating that atom C12 is *sp* hybridized. Atoms C2,C3,C4 and C5 are essentially coplanar. An intermolecular C54—H54···N13(1/2 - *x*, -1/2 + *y*, 1/2 - *z*) hydrogen bond is found in the crystal structure. In addition, a C56—H56···π(1/2 - *x*, 1/2 + *y*, 1/2 - *z*) interaction involving the phenyl ring (C51—C56) at position 5  is also found.

### Experimental

To a mixture of benzyl cyanide (1.12 ml, 0.01 mol) and potassium hydroxide (0.66 g, 0.01 mol) in 50 ml ethanol, *trans*-cinnamaldehyde (1.3 ml, 0.01 mol) was added and the solution was stirred for five minutes at room temperature. The solid obtained was separated, dried and then recrystallized from absolute ethanol. The yield of isolated product was 1.81 g (82%).

### Refinement

H atoms were positioned geometrically and allowed to ride on their parent atoms, with C—H = 0.95 Å; *U*_iso_(H) = 1.2*U*_eq_(C).

### Crystal data

**Table:**

C_17_H_13_N	*F*(000) = 976
*M**_r_* = 231.28	*D*_x_ = 1.251 Mg m^−^^3^
Monoclinic, *C*2/*c*	Melting point: 440 K
Hall symbol: -C 2yc	Cu *K*α radiation, λ = 1.54178 Å
*a* = 16.9390 (5) Å	Cell parameters from 4071 reflections
*b* = 7.5869 (2) Å	θ = 4.6–74.1°
*c* = 19.3809 (6) Å	µ = 0.56 mm^−^^1^
β = 99.521 (3)°	*T* = 110 K
*V* = 2456.42 (13) Å^3^	Prism, colourless
*Z* = 8	0.53 × 0.36 × 0.29 mm

### Data collection

**Table:**

Oxford Diffraction Xcalibur Ruby Gemini diffractometer	2420 independent reflections
Radiation source: Enhance (Cu) X-ray Source	2322 reflections with *I* > 2σ(*I*)
graphite	*R*_int_ = 0.014
Detector resolution: 10.5081 pixels mm^-1^	θ_max_ = 74.1°, θ_min_ = 4.6°
ω scans	*h* = −20→15
Absorption correction: multi-scan (*CrysAlis PRO*; Oxford Diffraction, 2009)	*k* = −8→9
*T*_min_ = 0.713, *T*_max_ = 1.000	*l* = −23→23
4517 measured reflections	

### Refinement

**Table:**

Refinement on *F*^2^	Primary atom site location: structure-invariant direct methods
Least-squares matrix: full	Secondary atom site location: difference Fourier map
*R*[*F*^2^ > 2σ(*F*^2^)] = 0.044	Hydrogen site location: inferred from neighbouring sites
*wR*(*F*^2^) = 0.121	H-atom parameters constrained
*S* = 1.02	*w* = 1/[σ^2^(*F*_o_^2^) + (0.0729*P*)^2^ + 1.834*P*] where *P* = (*F*_o_^2^ + 2*F*_c_^2^)/3
2420 reflections	(Δ/σ)_max_ = 0.001
163 parameters	Δρ_max_ = 0.34 e Å^−^^3^
0 restraints	Δρ_min_ = −0.22 e Å^−^^3^

### Special details

**Table:**

Geometry. Bond distances, angles *etc*. have been calculated using the rounded fractional coordinates. All su's are estimated from the variances of the (full) variance-covariance matrix. The cell e.s.d.'s are taken into account in the estimation of distances, angles and torsion angles
Refinement. Refinement of *F*^2^ against ALL reflections. The weighted *R*-factor *wR* and goodness of fit *S* are based on *F*^2^, conventional *R*-factors *R* are based on *F*, with *F* set to zero for negative *F*^2^. The threshold expression of *F*^2^ > 2σ(*F*^2^) is used only for calculating *R*-factors(gt) *etc*. and is not relevant to the choice of reflections for refinement. *R*-factors based on *F*^2^ are statistically about twice as large as those based on *F*, and *R*- factors based on ALL data will be even larger.

### Fractional atomic coordinates and isotropic or equivalent isotropic displacement parameters (Å^2^)

**Table:**

	*x*	*y*	*z*	*U*_iso_*/*U*_eq_	
N13	0.26391 (6)	0.47605 (14)	−0.01895 (5)	0.0260 (3)	
C2	0.40160 (7)	0.32134 (15)	0.01356 (6)	0.0195 (3)	
C3	0.42079 (7)	0.25057 (15)	0.07831 (6)	0.0211 (3)	
C4	0.37331 (7)	0.25955 (15)	0.13347 (6)	0.0209 (3)	
C5	0.39654 (7)	0.17874 (15)	0.19524 (6)	0.0214 (3)	
C12	0.32517 (7)	0.40883 (15)	−0.00422 (6)	0.0204 (3)	
C21	0.45270 (7)	0.31560 (14)	−0.04165 (6)	0.0189 (3)	
C22	0.42867 (7)	0.40207 (16)	−0.10539 (6)	0.0224 (3)	
C23	0.47643 (7)	0.39861 (16)	−0.15733 (6)	0.0250 (3)	
C24	0.54848 (7)	0.30767 (16)	−0.14678 (6)	0.0245 (3)	
C25	0.57313 (7)	0.22103 (16)	−0.08366 (6)	0.0242 (3)	
C26	0.52583 (7)	0.22532 (16)	−0.03154 (6)	0.0224 (3)	
C51	0.35609 (6)	0.18244 (15)	0.25646 (6)	0.0194 (3)	
C52	0.37767 (7)	0.05962 (15)	0.31035 (6)	0.0212 (3)	
C53	0.34045 (7)	0.05941 (15)	0.36908 (6)	0.0222 (3)	
C54	0.28173 (7)	0.18303 (16)	0.37571 (6)	0.0230 (3)	
C55	0.25983 (7)	0.30566 (16)	0.32272 (6)	0.0240 (3)	
C56	0.29636 (7)	0.30601 (16)	0.26368 (6)	0.0219 (3)	
H3	0.47024	0.18917	0.08834	0.0254*	
H4	0.32454	0.32395	0.12601	0.0250*	
H5	0.44452	0.11182	0.19968	0.0257*	
H22	0.37921	0.46385	−0.11333	0.0269*	
H23	0.45960	0.45882	−0.20022	0.0300*	
H24	0.58077	0.30461	−0.18245	0.0294*	
H25	0.62245	0.15873	−0.07614	0.0291*	
H26	0.54331	0.16626	0.01150	0.0269*	
H52	0.41827	−0.02457	0.30665	0.0254*	
H53	0.35524	−0.02567	0.40485	0.0266*	
H54	0.25673	0.18379	0.41613	0.0276*	
H55	0.21948	0.39005	0.32695	0.0288*	
H56	0.28080	0.39060	0.22787	0.0263*	

### Atomic displacement parameters (Å^2^)

**Table:**

	*U*^11^	*U*^22^	*U*^33^	*U*^12^	*U*^13^	*U*^23^
N13	0.0243 (5)	0.0299 (6)	0.0246 (5)	0.0032 (4)	0.0064 (4)	0.0016 (4)
C2	0.0198 (5)	0.0191 (6)	0.0198 (5)	−0.0021 (4)	0.0038 (4)	−0.0030 (4)
C3	0.0205 (6)	0.0216 (6)	0.0214 (6)	−0.0006 (4)	0.0037 (4)	−0.0013 (4)
C4	0.0199 (5)	0.0219 (6)	0.0209 (6)	−0.0005 (4)	0.0037 (4)	−0.0009 (4)
C5	0.0194 (5)	0.0229 (6)	0.0220 (6)	0.0003 (4)	0.0034 (4)	−0.0009 (4)
C12	0.0243 (6)	0.0216 (6)	0.0161 (5)	−0.0030 (4)	0.0060 (4)	−0.0010 (4)
C21	0.0206 (5)	0.0175 (5)	0.0188 (5)	−0.0036 (4)	0.0035 (4)	−0.0034 (4)
C22	0.0221 (5)	0.0240 (6)	0.0210 (6)	0.0010 (4)	0.0034 (4)	−0.0015 (4)
C23	0.0291 (6)	0.0274 (6)	0.0187 (5)	−0.0008 (5)	0.0045 (4)	0.0001 (4)
C24	0.0267 (6)	0.0264 (6)	0.0228 (6)	−0.0032 (5)	0.0109 (5)	−0.0040 (4)
C25	0.0214 (6)	0.0243 (6)	0.0279 (6)	0.0004 (4)	0.0070 (5)	−0.0019 (5)
C26	0.0235 (6)	0.0230 (6)	0.0208 (6)	−0.0001 (4)	0.0036 (4)	0.0013 (4)
C51	0.0181 (5)	0.0215 (6)	0.0179 (5)	−0.0029 (4)	0.0010 (4)	−0.0009 (4)
C52	0.0188 (5)	0.0209 (5)	0.0228 (6)	0.0009 (4)	0.0002 (4)	0.0002 (4)
C53	0.0245 (6)	0.0219 (6)	0.0188 (5)	−0.0025 (4)	−0.0002 (4)	0.0037 (4)
C54	0.0250 (6)	0.0258 (6)	0.0190 (5)	−0.0039 (4)	0.0059 (4)	−0.0008 (4)
C55	0.0239 (6)	0.0248 (6)	0.0240 (6)	0.0036 (5)	0.0062 (4)	0.0005 (4)
C56	0.0229 (6)	0.0233 (6)	0.0192 (5)	0.0020 (4)	0.0023 (4)	0.0039 (4)

### Geometric parameters (Å, °)

**Table:**

N13—C12	1.1491 (16)	C53—C54	1.3885 (17)
C2—C3	1.3542 (16)	C54—C55	1.3899 (17)
C2—C12	1.4450 (17)	C55—C56	1.3880 (16)
C2—C21	1.4841 (17)	C3—H3	0.9500
C3—C4	1.4418 (17)	C4—H4	0.9500
C4—C5	1.3447 (16)	C5—H5	0.9500
C5—C51	1.4653 (16)	C22—H22	0.9500
C21—C22	1.3985 (16)	C23—H23	0.9500
C21—C26	1.4006 (17)	C24—H24	0.9500
C22—C23	1.3921 (17)	C25—H25	0.9500
C23—C24	1.3874 (17)	C26—H26	0.9500
C24—C25	1.3907 (16)	C52—H52	0.9500
C25—C26	1.3897 (17)	C53—H53	0.9500
C51—C52	1.4023 (16)	C54—H54	0.9500
C51—C56	1.4032 (16)	C55—H55	0.9500
C52—C53	1.3894 (16)	C56—H56	0.9500
			
N13···C12^i^	3.3510 (16)	C56···H22^vii^	3.1000
N13···C54^ii^	3.3396 (16)	H3···C26	2.6600
N13···C4^i^	3.4376 (15)	H3···H5	2.3500
N13···C54^iii^	3.3883 (15)	H3···H26	2.1000
N13···H54^ii^	2.8600	H4···C12	2.6100
N13···H54^iii^	2.6100	H4···C56	2.7900
N13···H25^iv^	2.8300	H4···H56	2.2800
N13···H22	2.8900	H5···H3	2.3500
C3···C25^v^	3.5805 (17)	H5···H52	2.4200
C3···C23^vi^	3.4028 (17)	H5···C52^x^	3.0800
C4···N13^i^	3.4376 (15)	H5···H5^x^	2.4700
C4···C24^vi^	3.5341 (17)	H5···H52^x^	2.5700
C12···N13^i^	3.3510 (16)	H22···N13	2.8900
C12···C25^vi^	3.5755 (17)	H22···C12	2.4700
C12···C12^i^	3.5296 (17)	H22···C55^ii^	2.8000
C21···C21^vi^	3.4853 (16)	H22···C56^ii^	3.1000
C23···C3^vi^	3.4028 (17)	H23···H23^xi^	2.5400
C24···C4^vi^	3.5341 (17)	H23···H24^xi^	2.5500
C25···C12^vi^	3.5755 (17)	H24···H23^xi^	2.5500
C25···C3^v^	3.5805 (17)	H25···N13^xii^	2.8300
C54···N13^vii^	3.3396 (16)	H25···H54^xiii^	2.6000
C54···N13^viii^	3.3883 (15)	H26···C3	2.7000
C2···H53^ix^	3.0900	H26···H3	2.1000
C3···H26	2.7000	H26···C53^x^	2.9000
C4···H56	2.7800	H52···H5	2.4200
C5···H55^viii^	2.9300	H52···H5^x^	2.5700
C12···H22	2.4700	H52···C23^xiv^	3.0500
C12···H4	2.6100	H53···C2^xiv^	3.0900
C21···H53^ix^	2.8400	H53···C21^xiv^	2.8400
C23···H52^ix^	3.0500	H54···N13^vii^	2.8600
C26···H3	2.6600	H54···N13^viii^	2.6100
C51···H55^viii^	2.9100	H54···H25^xv^	2.6000
C52···H5^x^	3.0800	H55···C5^iii^	2.9300
C52···H56^viii^	2.9600	H55···C51^iii^	2.9100
C53···H56^viii^	2.8500	H56···C4	2.7800
C53···H26^x^	2.9000	H56···H4	2.2800
C54···H56^viii^	3.0600	H56···C52^iii^	2.9600
C55···H22^vii^	2.8000	H56···C53^iii^	2.8500
C56···H4	2.7900	H56···C54^iii^	3.0600
			
C3—C2—C12	118.13 (11)	C3—C4—H4	119.00
C3—C2—C21	125.26 (11)	C5—C4—H4	119.00
C12—C2—C21	116.61 (10)	C4—C5—H5	116.00
C2—C3—C4	126.20 (11)	C51—C5—H5	116.00
C3—C4—C5	121.44 (11)	C21—C22—H22	120.00
C4—C5—C51	127.23 (11)	C23—C22—H22	120.00
N13—C12—C2	178.90 (13)	C22—C23—H23	120.00
C2—C21—C22	120.26 (10)	C24—C23—H23	120.00
C2—C21—C26	121.42 (10)	C23—C24—H24	120.00
C22—C21—C26	118.32 (11)	C25—C24—H24	120.00
C21—C22—C23	120.72 (11)	C24—C25—H25	120.00
C22—C23—C24	120.34 (11)	C26—C25—H25	120.00
C23—C24—C25	119.57 (11)	C21—C26—H26	120.00
C24—C25—C26	120.20 (11)	C25—C26—H26	120.00
C21—C26—C25	120.84 (11)	C51—C52—H52	120.00
C5—C51—C52	119.42 (10)	C53—C52—H52	120.00
C5—C51—C56	122.34 (10)	C52—C53—H53	120.00
C52—C51—C56	118.24 (10)	C54—C53—H53	120.00
C51—C52—C53	120.88 (11)	C53—C54—H54	120.00
C52—C53—C54	120.27 (11)	C55—C54—H54	120.00
C53—C54—C55	119.44 (11)	C54—C55—H55	120.00
C54—C55—C56	120.63 (11)	C56—C55—H55	120.00
C51—C56—C55	120.54 (11)	C51—C56—H56	120.00
C2—C3—H3	117.00	C55—C56—H56	120.00
C4—C3—H3	117.00		
			
C12—C2—C3—C4	−2.66 (18)	C22—C21—C26—C25	−0.32 (17)
C21—C2—C3—C4	177.83 (11)	C21—C22—C23—C24	0.57 (18)
C3—C2—C21—C22	−176.35 (12)	C22—C23—C24—C25	−0.55 (18)
C3—C2—C21—C26	3.30 (18)	C23—C24—C25—C26	0.10 (19)
C12—C2—C21—C22	4.13 (16)	C24—C25—C26—C21	0.34 (18)
C12—C2—C21—C26	−176.22 (11)	C5—C51—C52—C53	−179.94 (11)
C2—C3—C4—C5	177.44 (12)	C56—C51—C52—C53	0.50 (17)
C3—C4—C5—C51	177.45 (11)	C5—C51—C56—C55	−179.63 (11)
C4—C5—C51—C52	165.27 (12)	C52—C51—C56—C55	−0.08 (17)
C4—C5—C51—C56	−15.19 (19)	C51—C52—C53—C54	−0.85 (18)
C2—C21—C22—C23	179.52 (11)	C52—C53—C54—C55	0.76 (18)
C26—C21—C22—C23	−0.14 (17)	C53—C54—C55—C56	−0.34 (18)
C2—C21—C26—C25	−179.97 (10)	C54—C55—C56—C51	0.00 (18)

Symmetry codes: (i) −*x*+1/2, −*y*+1/2, −*z*; (ii) *x*, −*y*+1, *z*−1/2; (iii) −*x*+1/2, *y*+1/2, −*z*+1/2; (iv) *x*−1/2, *y*+1/2, *z*; (v) −*x*+1, −*y*, −*z*; (vi) −*x*+1, −*y*+1, −*z*; (vii) *x*, −*y*+1, *z*+1/2; (viii) −*x*+1/2, *y*−1/2, −*z*+1/2; (ix) *x*, −*y*, *z*−1/2; (x) −*x*+1, *y*, −*z*+1/2; (xi) −*x*+1, *y*, −*z*−1/2; (xii) *x*+1/2, *y*−1/2, *z*; (xiii) *x*+1/2, −*y*+1/2, *z*−1/2; (xiv) *x*, −*y*, *z*+1/2; (xv) *x*−1/2, −*y*+1/2, *z*+1/2.

### Hydrogen-bond geometry (Å, °)

**Table:**

*D*—H···*A*	*D*—H	H···*A*	*D*···*A*	*D*—H···*A*
C54—H54···N13^viii^	0.95	2.61	3.388 (2)	139
C56—H56···Cg1^iii^	0.95	2.83	3.657 (1)	146

Symmetry codes: (viii) −*x*+1/2, *y*−1/2, −*z*+1/2; (iii) −*x*+1/2, *y*+1/2, −*z*+1/2.
